# Enhancing bio-oil yield extracted from Egyptian castor seeds by using microwave and ultrasonic

**DOI:** 10.1038/s41598-023-31794-3

**Published:** 2023-03-21

**Authors:** El-Sayed G. Khater, Soha A. Abd Allah, Adel H. Bahnasawy, Hassan M. Abu Hashish

**Affiliations:** 1 Agricultural and Biosystems Engineering Department, Faculty of Agriculture, Benha University, P.O. Box 13736, Moshtohor, Toukh, Kalubia Egypt; 2 Mechanical Engineering Department, Engineering Research Division, National Research Centre, Giza, Egypt

**Keywords:** Ecology, Environmental sciences, Energy science and technology, Engineering

## Abstract

Energy scarcity and conventional energy problems are the main reason of finding a renewable source of energy which is cheap and environmental friendly, therefore, biodiesel production is one of the most promising solutions of this problem. Also, Egyptian castor is one of the most important crops for oil production compared with other commonly used oil crops. The main aim of this study is to enhance the production of bio-oil from Egyptian castor seeds by using microwave and ultrasonic as pre-treatments. To achieve that, the effects of extraction screw speed (20, 40 and 60 rpm) and temperature (100, 150, 200 and 250 °C) on oil extraction yield and quality, extraction energy requirements and extraction time and were studied. Also, the effect of pretreatment conditions of microwave at three levels of power (Low, Med and High) and different times (1, 2 and 3 min) and pretreatment condition ultrasonic with different temperatures (40, 60 and 80 °C) and different times (15, 30 and 45 min) for castor seeds before extraction with the optimum condition of the screw press on oil extraction yield from castor seeds, extraction energy, extraction time and quality of the oil extracted. The results indicate that the optimum conditions oil extraction by screw press were 200 °C extraction temperature and 60 rpm screw speed. It could be seen that the extraction oil yield, extraction energy requirements and extraction time were 35.59%, 18.68 and 1.86 min, respectively. Microwave pretreatments had better on oil yield and energy required for extraction compared to ultrasonic pretreatments, where, microwave pretreatments recorded high oil yield and lower energy requirements compared to the ultrasonic pretreatments. Oil yield ranged from 32.67 to 37.41% compared to 13.29 to 39.83% in literature. The time required for extraction was ranged from 1.77 to 2.00 and 1.79 to 2.21 min for microwave and ultrasonic pretreatments, respectively. The pretreatment improved properties of the extracted oil.

## Introduction

Industries are consuming energy less than that of buildings. Transportation comes in the third level in consuming energy after both buildings and transportation. Agriculture, fishing and forestry activities are the low level of energy consumption. The energy consumption increased to 9.1 Giga ton in all these activities during the year of 2019^1^.

Fuel consumption for transportation increased twice during the lost decades, due to the increase in population, urbanization, and global mobility. This sector consumed about 28% of total energy demand. Passengers movements and goods consumed about 70% of the total energy consumed in transportation. Due to using the oil-based fuels in about 95% of the energy used for transportation, environment and energy security are affected deeply ^2^.

Energy scarcity is a very serious problem. Egypt’s energy balance in 2022 indicates that the industrial sector consume the largest of final energy consumption (34.2%), followed by transportation (24.2%), residential buildings (18.8%) and agriculture and mining (4.7%), that makes 81.9% of the total consumption^3^. Todays, the fuel price crisis with the awareness problem is the most effective factors on the structure of energy usage all over the world. Greater efforts have been devoted by the researchers to develop a renewable energy. Renewable energy is the key factor for the future not only for Egypt but also for the whole-world^4^. Therefore, using biodiesel as alternative resource of energy reduces pollutant and potential carcinogens. It has low emissions because the organic carbon used in photogeneration. Also, it does not increase the levels of carbon dioxide in the atmosphere^5,6^.

Plants such as soybean, corn, palm, and castor oils are used to produce biodiesel by reaction with catalyzed alcohols. These oils may be edible or nonedible oils. Using nonedible oils in biodiesel is better for helping in food deficit problems. The prices of biodiesel are depending on the cost of crude oil (75%). Using low prices raw materials reduces the price of biodiesel. In Egypt, the price of castor oil is cheaper than Jatropha oil^7^. The difference in oil price that obtained from castor is less (by 15%) than that of Jatropha in Egypt.

Castor plant *(Ricinus communis)* is a perennial shrub that can grow up to 3–5 m long and found across all the tropical and subtropical areas of the globe. India ranks first in the world with an average production of around 10^6^ t of castor seed and 3.5–5.0 × 10^5^ t of castor oil^8,9^. Castor is very important crop and its seeds are used for oil production as a source of biodiesel. It is grown on marginal lands with low water requirements. It is pest-resistant and drought tolerant. Castor seeds contain 40–55%, oil which is very high compared to other commonly used crops such as soybean (15–20%), sunflower (25–35%), rapeseed (38–46%), Palm (30–60%). It is characterized by its high Ricinoleic acid content which is proper for industrial rather than food applications. Castor is considered a sustainable source of biodiesel as the production cost of castor seeds is production of castor seeds is less than that of jatropha, soybean and rapeseed ^5,7,10^.

Oil is extracted by several methods such as mechanical, chemical and enzymatic methods. Using organic solvent in biodiesel extraction is considered the best way but it affects the environment because of the emission of volatile organic compounds during process. From the practical point of view, the mechanical extraction is the best methods because it is safe, sample and lower in cost^6^. More oil can be extracted by using engine driven screw piston (68–80%) compared to 60–65% when using engine driven raw piston. Pretreatment of crops such as frying increased the oil yield up to 89 to 91% after double-pass^6,11^.

Microwave pretreatments of oil extraction improve the oil yield and quality with lower energy consumption, safer time and lower solvent levels compared to conventional methods^12^. Microwave pretreatment oil has similar properties to conventionally extracted oil such as acidity value, peroxide value and oil composition^13^. Microwave radiation causes fraction which leads to heating. Fats have low specific heat which makes them vulnerable to this radiation and creates permanent pores in seeds resulting in higher yields and improved quality. Microwave pretreatments as an alternative to conventional method with faster processing, lower energy consumption and shorter exposure time^14^.

The application of ultrasonic pretreatment in the oil extraction might improve the oil extraction efficiency, the extracted oil’s quality, and the extracted phenolic compounds content^15^. Using the traditional methods of oil extraction result in low oil yield, low extraction efficiency, high energy consumption and low quality of oil extracted, therefore, using pretreatments of seeds is the main idea of this work to improve the energy consumption, finally, improve the oil quality. Microwave and ultrasonic pretreatments are the most commonly used pretreatments in oil extraction for seeds.

## Experimental Procedures

The main experiment was carried out in National Research Centre, Giza, Egypt. During summer season of 2021.

### Egyptian castor seeds

**Figure 1:**
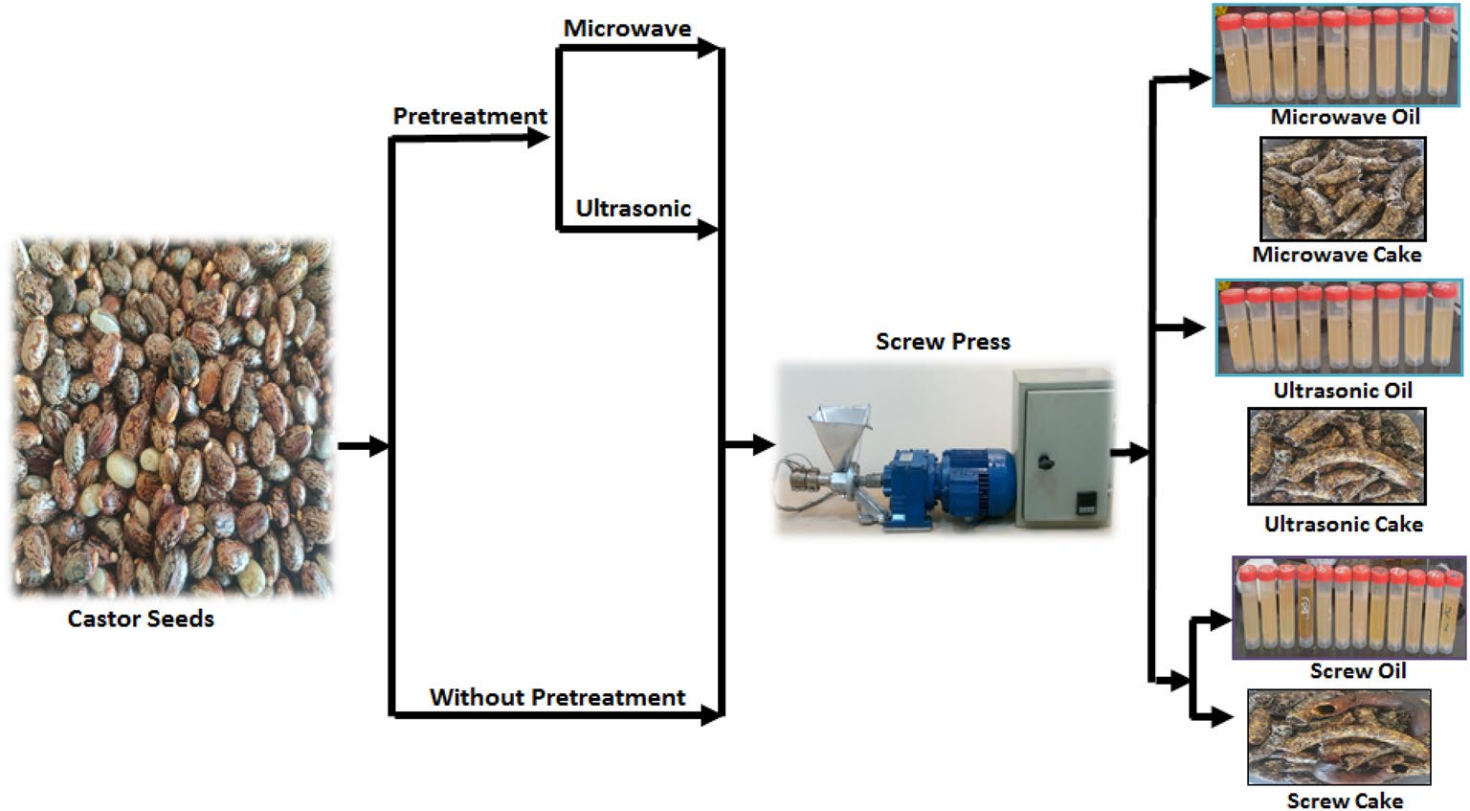
Schematic diagram of oil extraction by screw press from Egyptian castor seeds.

Egyptian castor seeds were obtained from a privet farm in Sinai Governorate. Castor seeds have been used under the permission of Benha University regulations. Plant materials are complied with the local and national regulations. A lipid analysis was performed showing the percentage of oil in the seeds. The bio-oil was extracted from the Egyptian castor seeds by a screw press without pre-treatments and pre-treatments were accomplished using Microwave and ultrasonic pretreatments to enhance the production of bio-oil from castor seeds. The schematic diagram in Fig. 1 shows the sequence of oil extraction by screw press from castor seeds.

### Mechanical screw pressing extraction method

**Figure 2:**
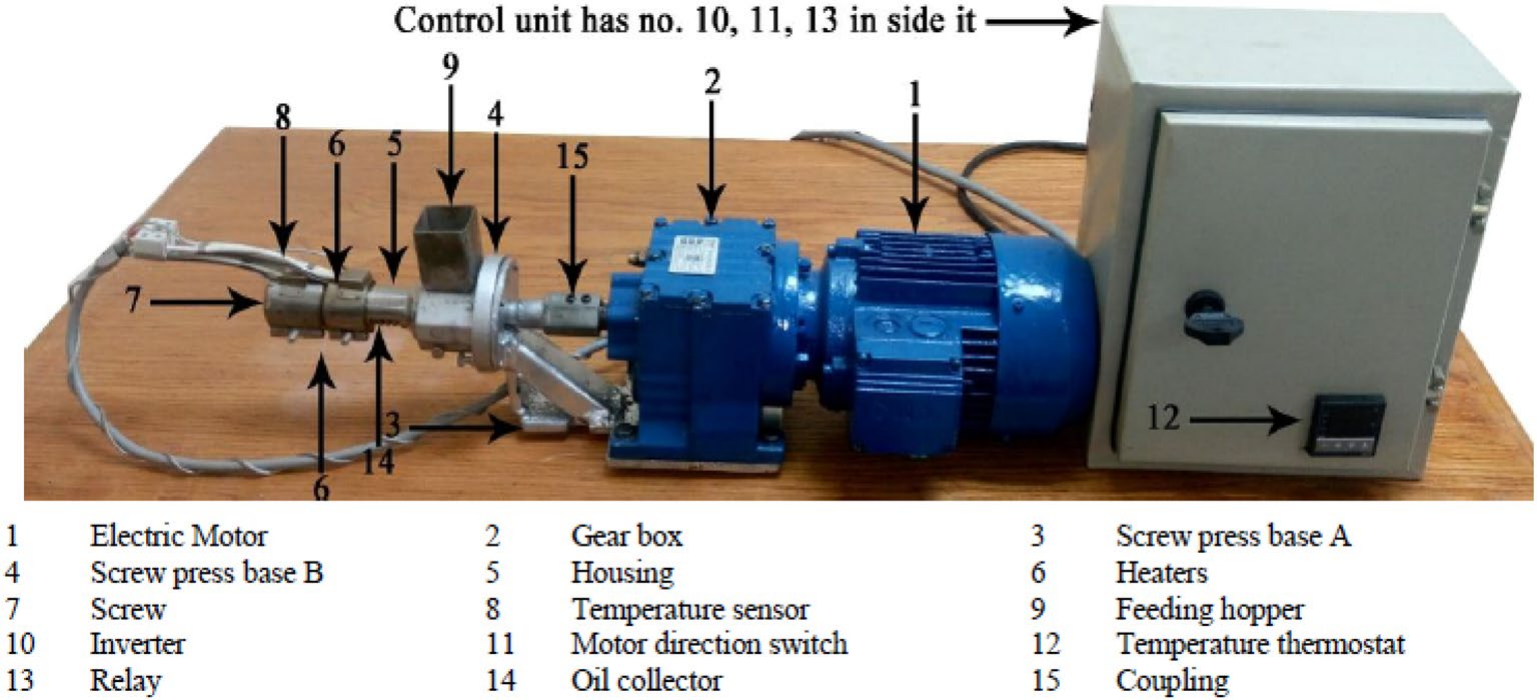
Photograph of the screw press.

The oil extraction was performed using a specially designed laboratory scale mechanical screw press in the National Research Center was used and whose specifications are mentioned its parts explained by^16^. A photograph of the screw press and its are shown in Fig. 2.

The screw press was used in oil extraction as studies have proven its success and efficient in extraction. Castor seeds were pressed at temperatures of 50 to 100 °C and speed of 40, 80 and 120 rpm, so the temperatures were raised and the parameters and conditions were chosen to suit the nature and characteristics of the castor seeds. Therefore, the experiments were conducted in factorial arrangement, with three speeds of 40, 60 and 80 rpm, at extraction temperatures of 100, 150, 200 and 250 °C where three replicates were made for an experiment. The oil yield and cake were weighing. Power and energy consumption were measured for each treatment by energy meter machine, and the time required for pressing for each treatment was also recorded.

### Pretreatments

#### Microwave pretreatment

**Figure 3:**
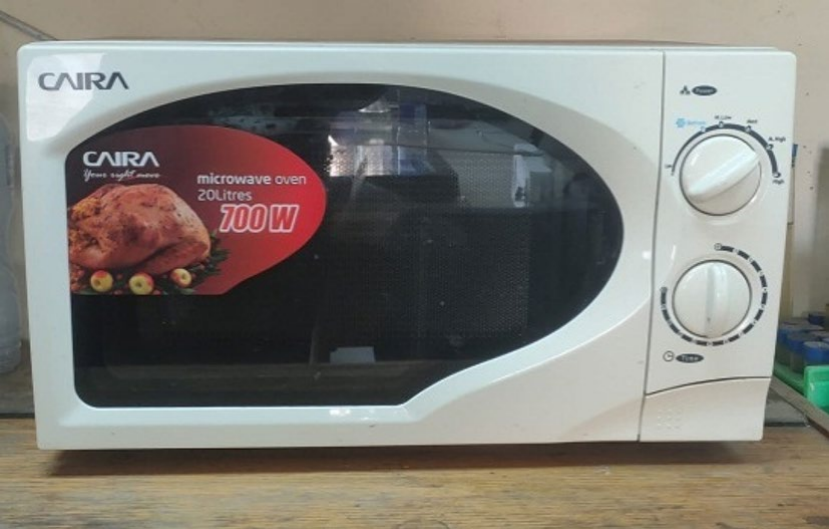
Microwave machine.

A microwave with power 700 W and 20 L capacity was used as shown in Fig. 3. The CARIA is owned by the Benha University and this work is done under the rules of this university. The treatments were selected according to^14^. The castor seeds were heated in the microwave and the samples were weighed before and after heating to measure the loss in moisture. The samples were heated in the microwave for 1, 2 and 3 min at three levels power low, medium and high and then the samples were pressed in the screw press immediately after heating in the microwave at the optimum parameters that were determined in the screw press. The energy required for heating in the microwave and the power for each samples were measured by energy meter machine and then both the oil and cake were weighed.

#### Ultrasonic pretreatment

**Figure 4:**
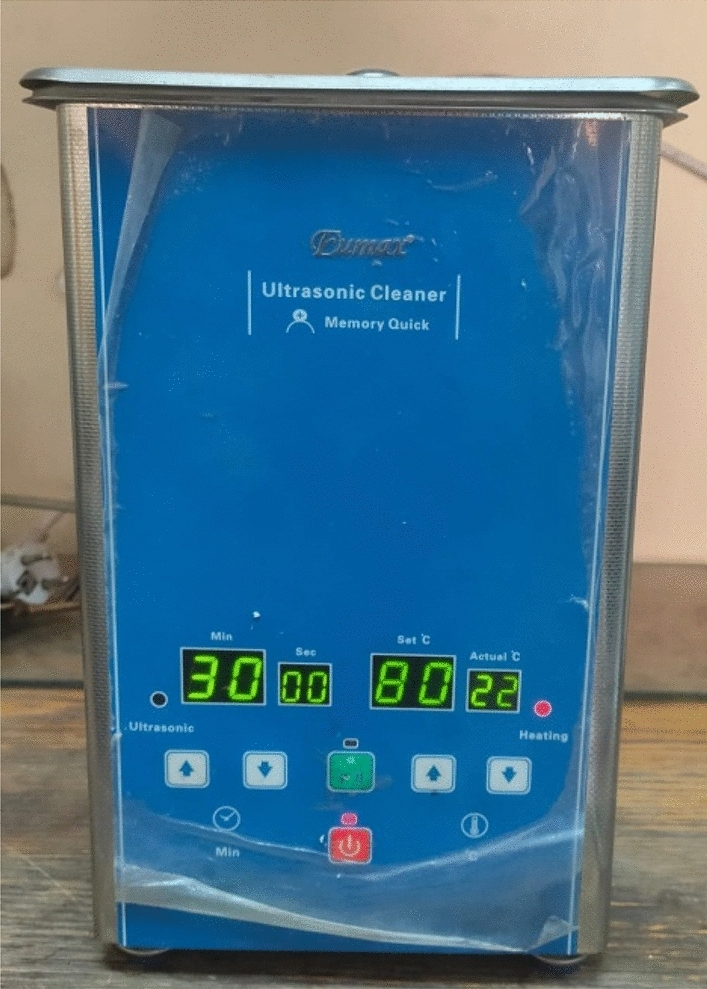
Ultrasonic cleaner.

Ultrasonic device (Model: UD50Sh-2.5LQ, Ultrasonic power: 50W, Total consumption power: 160W and Power supply: 220 VAC, 50 Hz) was used as shown in Fig. 4. The Eumax is owned by the Benha University and this work is done under the rules of this university. Three different exposure times (15, 30 and 45 min) and temperatures (40, 60 and 80 °C) were used. The samples were weighed before and after heating in to measure moisture loss, then the samples were transferred to the screw press at the optimum parameters that were determined in the screw press. Three replicates were made for the experiment. The energy consumption for heating in the ultrasonic and the power for each sample were measured, and then both the oil and cake were weighed.

### Oil quality measurements

The gas chromatography (GC) test is a technique used to measure the fatty acid composition in an oil sample which allows separation of mixtures based on their boiling point^17^. The fatty acid composition was determined by the transmethylation of the fatty chains to fatty acid methyl esters (FAMEs) according to the modified method by^18^. The oil samples of 0.2 g were mixed with 6 mL of methanolic sodium hydroxide solution. The mixture is refluxed for 10 min and then, adding HCl (30 mL) and 20 mL methanol and refluxed for another 10 min, then adding 10 mL hexane, and refluxed for another 2 min, and then left to cool. Finally, adding distilled water (10 mL) and poured into separating tunnel. The upper layer is collected and dried with calcium chloride. The FAMEs were separated with an HP 6890 plus gas chromatography (Hewlett Packard, USA), using a capillary column Supelco™ SP-2380 (60 m × 0.25 mm × 0.20 μm), (Sigma-Aldrich, USA), Detector (FID) and the injector and detector temperature was 250 °C. The column temperature was 140 °C (held for 5 min) and rose to 240 °C, at rate of 4 °C/min, and held at 240 °C for 10 min. The carrier gas was helium at flow rate 1.2 mL min^-1^. Sample volume was 1µL (in *n-*hexane) and injected through a split injector at splitting ratio of 100:20. FAMEs were identified by comparing their relative and absolute retention times to those authentic standards of FAMEs (Supelco™ 37component FAME mix). The fatty acid composition was reported as a relative percentage of the total peak area^18^.

Saponification number, Acid value and molecular weight of oil were determined according to^19^. The acid value of oil was determined by titrating of solution of oil in diethyl ether with alcoholic solution of sodium or potassium hydroxide. Each 1 g of oil is expressed by the amount of KOH which is used to neutralize the oil.

## Results and discussion

### Screw press conditions

#### Effect of extraction screw speed and temperature on oil extracted yield

**Figure 5:**
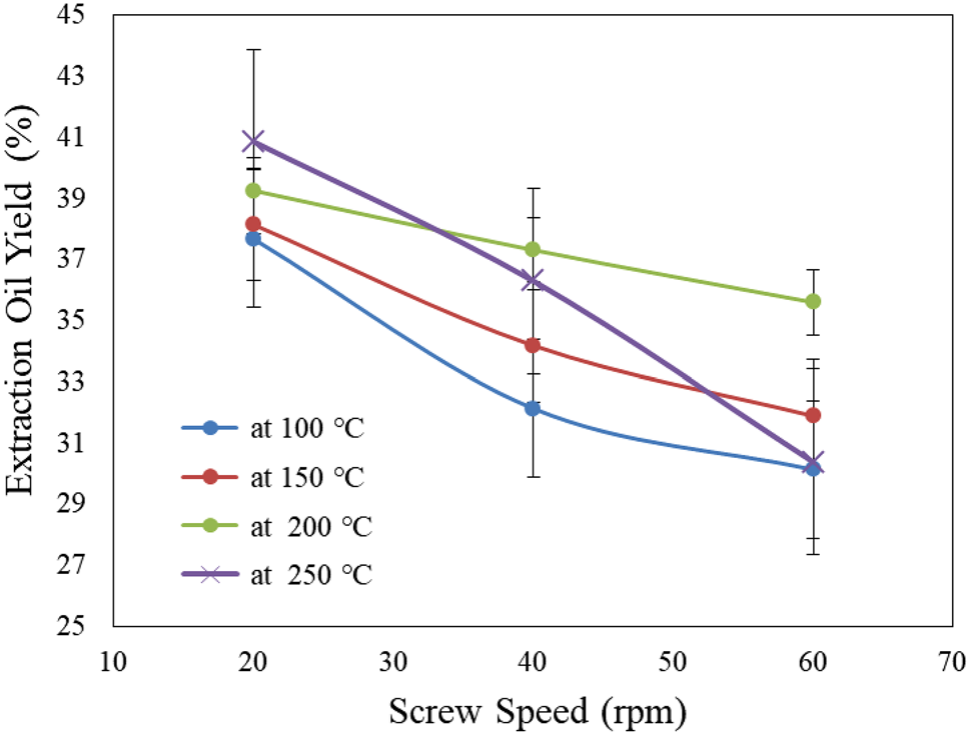
Effect of extraction screw speed and temperature on oil extraction yield castor.

Figure 5 shows the effect of extraction screw speed (20, 40 and 60 rpm) and temperature (100, 150, 200 and 250 °C) on oil yield from castor seeds. The results indicate that the oil yield decreases with increasing screw speed and decreasing temperature. It could be seen that the oil yield decreased from 37.66 to 30.13 (by 19.99%), 38.13 to 31.88 (by 16.39%), 39.24 to 35.59 (by 9.30%) and 40.85 to 30.37 (by 25.65%) % when the screw speed increased from 20 to 60 rpm, respectively, for 100, 150, 200 and 250 °C. The results also indicate that the highest value of decreasing of oil extraction yield (25.65%) was found for 250 °C extraction temperature. This was may be due to that with higher temperature, the oil exit from the cell easy^17^.

Also, the high temperature causes evaporate the oil from the cell, which leads to a decrease of oil yield. These results agreed with those obtained by^16^ whose found the lowest value of oil yield extraction was found the highest value of extraction temperature. The results indicate that the maximum oil production is about 40.84% by the screw press which is equivalent to 83.41% by mass fraction of oil in seeds in comparison percentage oil 48.96% in seeds obtained at an engine speed of 20 rpm and preheating temperature of 250 °C. These results agreed with those obtained by^1^. However, in these extraction conditions, much time is consumed with higher energy. The maximum yield of oil (40.85%) was obtained at temperatures in the range of 250 °C at speed of 20 rpm.

#### Effect of extraction screw speed and temperature on extraction energy consumption

**Figure 6:**
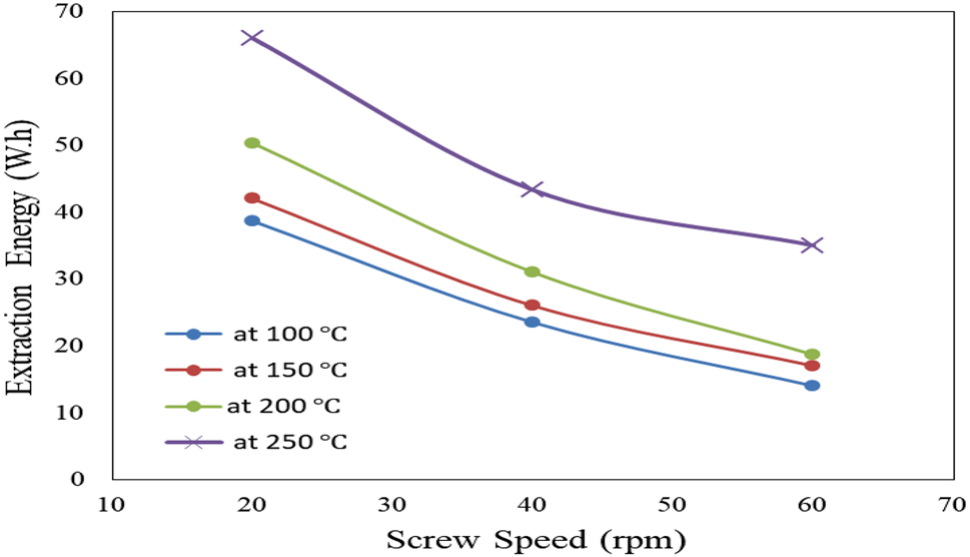
Effect of extraction screw speed and temperature on extraction energy.

Figure 6 shows the effect of extraction screw speed (20, 40 and 60 rpm) and temperature (100, 150, 200 and 250 °C) on oil extraction energy consumption from castor seeds. The results indicate that the extraction energy consumption decreases with increasing screw speed and decreasing temperature. It could be seen that the extraction energy consumption decreased from 38.67 to 14.00 (by 63.80%), 42.00 to 17.00 (by 59.52%), 50.33 to 18.67 (by 62.90%) and 66.00 to 35.00 (by 46.97%) W.h when the screw speed increased from 20 to 60 rpm for 100, 150, 200 and 250 °C, respectively. The results also indicate that the highest value of extraction energy consumption (66.00 W.h) was found of 250 °C extraction temperature and 20 rpm screw speed, while, the lowest value of extraction energy consumption (14.00 W.h) was found of 100 °C extraction temperature and 60 rpm screw speed. These results agreed with those obtained by^20^.

#### Effect of extraction screw speed and temperature on extraction time

**Figure 7:**
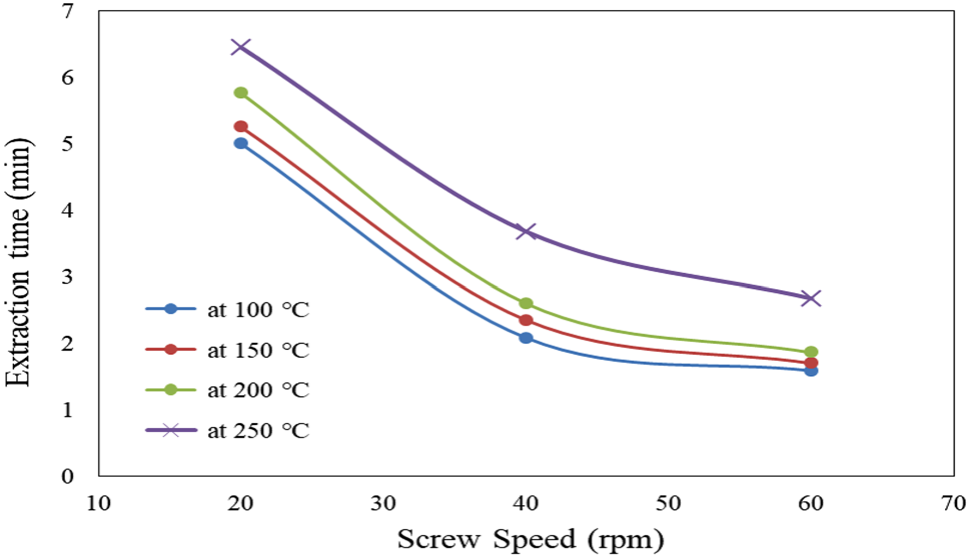
Effect of extraction screw speed and temperature on extraction time.

Figure 7 shows the effect of extraction screw speed (20, 40 and 60 rpm) and temperature (100, 150, 200 and 250 °C) on oil extraction time from castor seeds. The results indicate that the extraction time decreases with increasing screw speed and decreasing temperature. It could be seen that the extraction time decreased from 5.00 to 1.59 (by 68.20%), 5.25 to 1.70 (by 67.62%), 5.76 to 1.86 (by 67.71%) and 6.45 to 2.68 (by 58.45%) min when the screw speed increased from 20 to 60 rpm for 100, 150, 200 and 250 °C, respectively. The results also indicate that the highest value of extraction time (6.45 min) was found of 250 °C extraction temperature and 20 rpm screw speed, while, the lowest value of extraction time (1.59 min) was found of 100 °C extraction temperature and 60 rpm screw speed. These results agreed with those obtained by^21^.

The results indicate that the optimum conditions of screw press for extracted caster seeds were 200 °C extraction temperature and 60 rpm screw speed. It could be seen that the extraction oil yield, extraction energy and extraction time were 35.59%, 18.68 and 1.86 min, respectively.

### Pretreatment condition

#### Effect of pretreatment conditions (microwave and ultrasonic) on oil extraction yield

**Figure 8:**
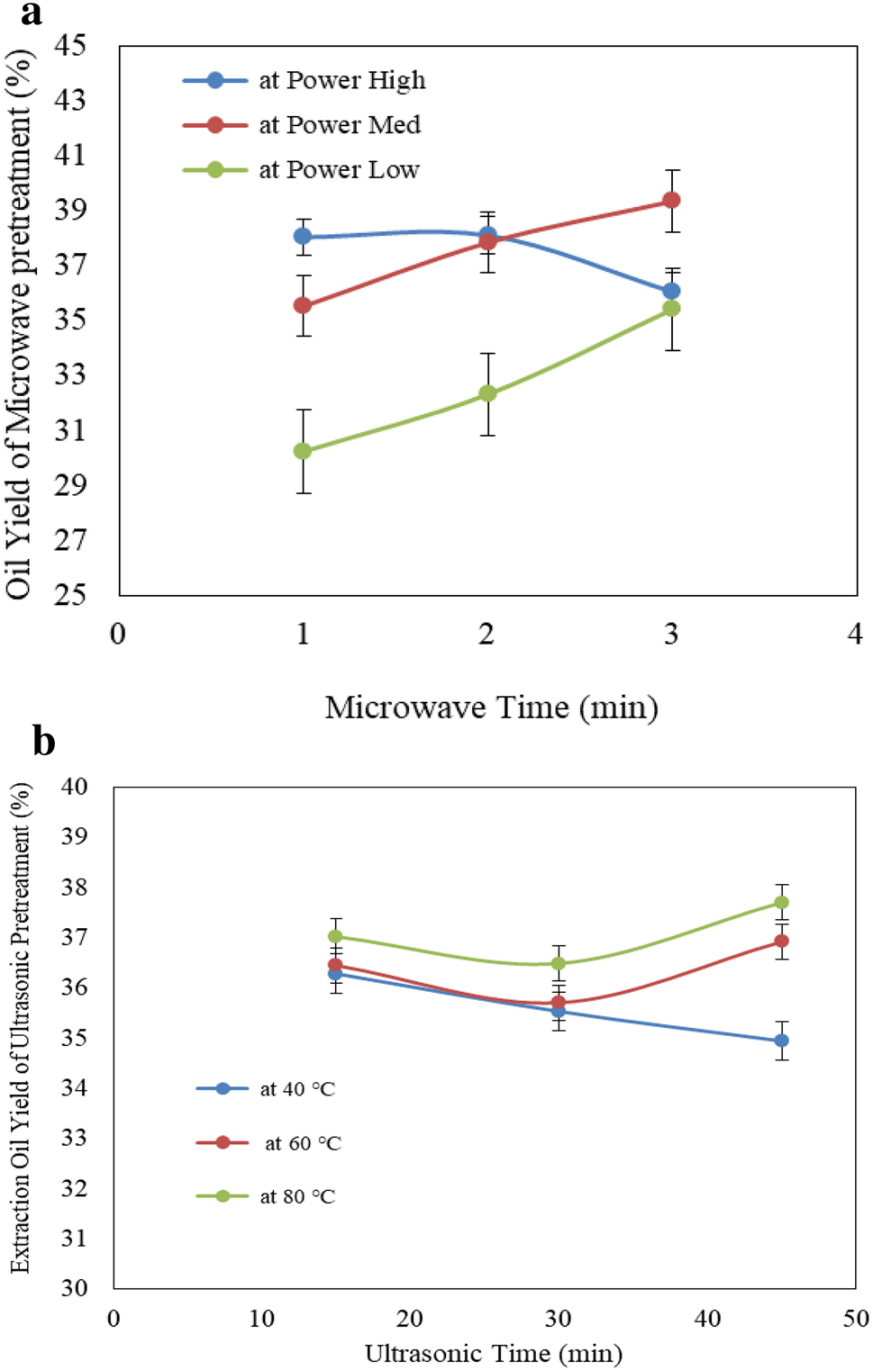
(**a**) Effect of microwave condition on oil extraction yield. (**b**) Effect of ultrasonic condition on oil extraction yield.

Figure 8a shows the effect of microwave power level (low, medium and high) and operating time (1, 2 and 3 min) on oil extraction yield from castor seeds. The results indicate that the oil extraction yield increases with increasing operating time for low and medium microwave power levels and it decreases with increasing operating time for high microwave power level. It could be seen that the oil extraction yield increased from 30.24 to 35.43 and 35.54 to 39.36% when the operating time increased from 1 to 3 min, respectively for low and medium microwave power levels, respectively. Meanwhile, it was decreased from 38.05 to 36.06% when the operating time increased from 1 to 3 min, respectively, for high microwave power level. This is due to the high microwave power level causes evaporation oil moisture from the cell, which leads to a decrease of oil yield. The results also indicate that the highest value of oil extraction yield (39.36%) was found of 3 min operating time and medium microwave power level compared non-treatment, the oil extraction yield was 35.59% at the same conditions (200 °C extraction temperature and 60 rpm screw speed).

Figure 8b shows the effect of ultrasonic temperature (40, 60 and 80 °C) and operating time (15, 30 and 45 min) on oil extraction yield from castor seeds. The results indicate that the oil extraction yield values were 36.26, 35.54 and 34.95, 36.46, 35.71 and 36.93 and 37.03, 36.50 and 37.71% at 15, 30 and 45 min operating time, respectively for 40, 60 and 80 °C ultrasonic temperatures. The results also indicate that the highest value of oil extraction yield (37.03%) was found of 15 min operating time and 80 °C ultrasonic temperature compared non-treatment.

The results also indicate that the oil extraction yield from castor seeds by using microwave pretreatment was higher than those of ultrasonic pretreatment for medium and high power levels and medium and high temperature, while the oil extraction yield from caster seeds by using microwave pretreatment was lower than those of ultrasonic pretreatment for low power levels and low temperatures. It could be seen that the oil extraction yield values were 32.67, 37.59 and 37.41% for low, medium and high microwave power levels, respectively, but they were 35.59, 36.37 and 37.08% for 40, 60 and 80 °C ultrasonic temperature, respectively. Also, the oil extraction yield form castor seeds by using microwave pretreatment were higher than those of ultrasonic pretreatment for medium and high operating time, while the oil extraction yield for extracted castor seeds from castor seeds by using microwave pretreatment was lower than those of ultrasonic pretreatment for low operating time. It could be seen that the oil extraction yield values were 34.61, 36.10 and 36.95% for 1, 2 and 3 min microwave operating time, respectively, but they were 36.59, 35.92 and 36.53% for 15, 30 and 45 min ultrasonic operating time, respectively. Generally, the oil yield from castor seeds ranged from 32.67 to 37.41% compared to 13.29 to 39.83% in literature^22^.

#### Effect of pretreatment condition (microwave and ultrasonic) on extraction energy consumption

**Figure 9:**
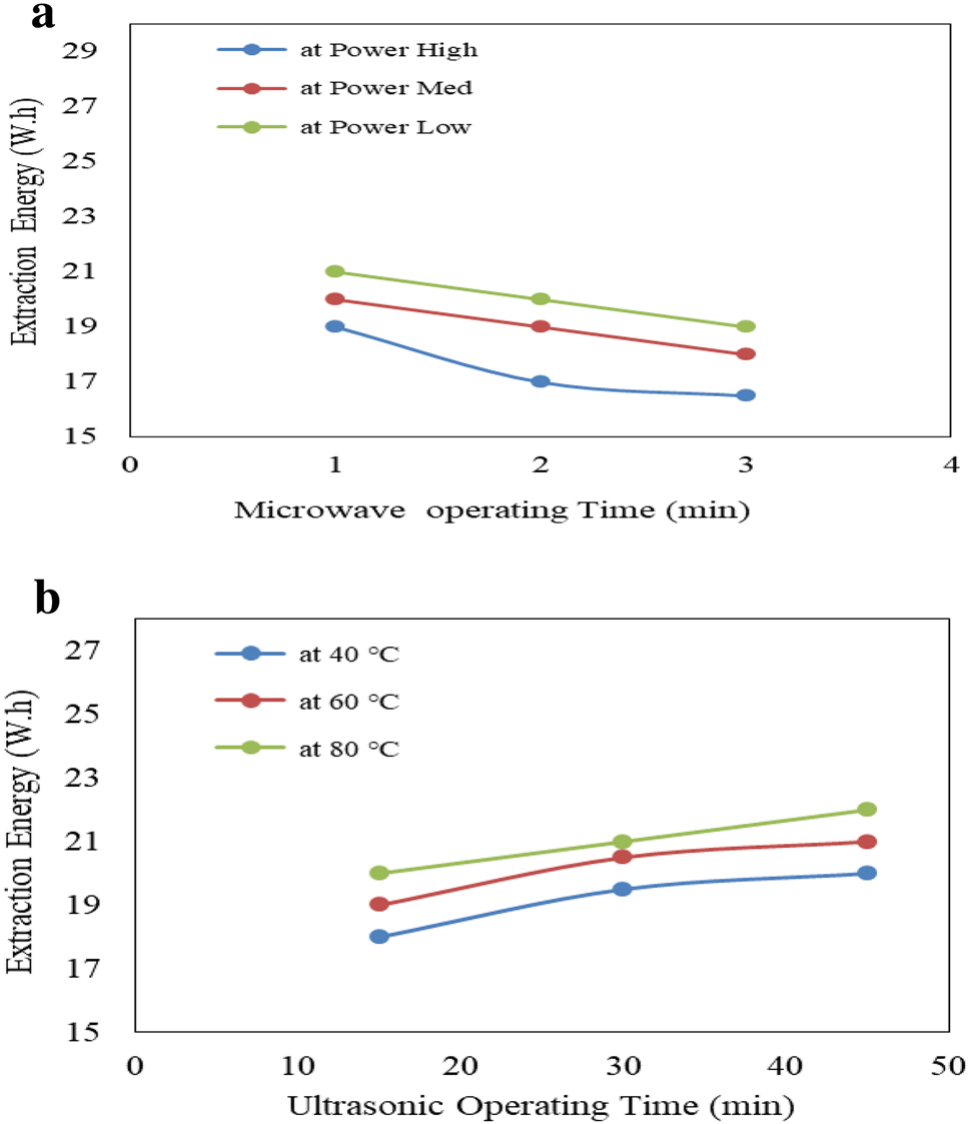
(**a**) Effect of microwave condition on oil extraction energy. (**b**) Effect of ultrasonic condition on extraction energy.

Figure 9a shows the effect of microwave power (low, medium and high) and operating time (1, 2 and 3 min) on extraction energy consumption from castor seeds. The results indicate that the extraction energy consumption decreases with increasing microwave power level and operating time. It could be seen that the extraction energy consumption decreased from 21.0 to 19.0, 20.0 to 18.0 and 19.0 to 16.5 W.h when the operating time increased from 1 to 3 min, respectively for low, medium and high microwave power levels. On the other hand, the energy consumed by microwave increases with increasing microwave power level and operating time. It could be seen that the energy consumed by microwave increased from 4.33 to 10.67, 14.00 to 40.00 and 17.67 to 51.67 W.h when the operating time increased from 1 to 3 min, respectively for low, medium and high microwave power levels. The results also indicate that the highest value of extraction energy (21.0 W.h) was found of 1 min operating time and low microwave power level. While, the lowest value of extraction energy (16.5 W h) was found of 3 min operating time and high microwave power level.

Figure 9b shows the effect of ultrasonic temperatures (40, 60 and 80 °C) and operating time (15, 30 and 45 min) on extraction energy consumption from castor seeds. The results indicate that the extraction energy consumption increases with increasing ultrasonic temperature and operating time. It could be seen that the extraction energy consumption increased from 18.0 to 20.0, 19.0 to 21.0 and 20.0 to 22.0 W h when the operating time increased from 15 to 45 min, respectively for 40, 60 and 80 °C ultrasonic temperature. While, the energy consumed by ultrasonic increases with increasing ultrasonic temperature and operating time. It could be seen that the energy consumed by ultrasonic increased from 11.00 to 34.95, 12.40 to 36.33 and 16.33 to 38.00 W h when the operating time increased from 15 to 45 min, respectively for 40, 60 and 80 °C ultrasonic temperature. The results also indicate that the highest value of extraction energy consumption (38.0 W h) was found of 45 min operating time and 80 °C ultrasonic temperature. While, the lowest value of extraction energy consumption (18.0 W h) was found of 15 min operating time and 40 °C ultrasonic temperature.

The results also indicate that the extraction energy consumption from castor seeds by using microwave pretreatment were lower than those of ultrasonic pretreatment. It could be seen that the extraction energy consumption from castor seeds were 20.0, 19.0 and 17.5 W h for low, medium and high microwave power levels, respectively, but they were 19.17, 20.17 and 21.00 W.h for 40, 60 and 80 °C ultrasonic temperature, respectively. Also, the extraction energy consumption from castor seeds by using microwave pretreatment was higher than those of ultrasonic pretreatment for medium and high operating time, while the extraction energy by using microwave pretreatment was lower than those of ultrasonic pretreatment for low operating time. It could be seen that the extraction energy consumption were 20.0, 19.0 and 17.33 W h for 1, 2 and 3 min microwave operating time, respectively, but they were 19.0, 20.3 and 21.0 W h for 15, 30 and 45 min ultrasonic operating time, respectively.

#### Effect of pretreatment conditions (microwave and ultrasonic) on extraction time

**Figure 10:**
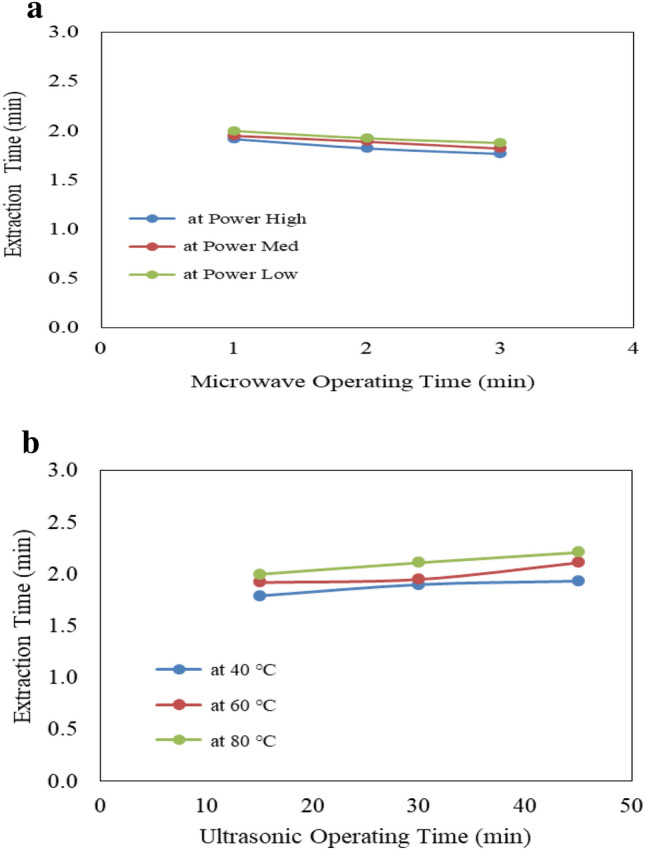
(**a**) Effect of microwave condition on oil extraction time. (**b**) Effect of ultrasonic condition on oil extraction time.

Figure 10a shows the effect of microwave power level (low, medium and high) and operating time (1, 2 and 3 min) on extraction time from castor seeds. The results indicate that the extraction time decreases with increasing microwave power level and operating time. It could be seen that the extraction time decreased from 2.00 to 1.88, 1.95 to 18.82 and 1.92 to 1.77 min when the operating time increased from 1 to 3 min, respectively for low, medium and high microwave power levels. The results also indicate that the highest value of extraction time (2.0 min) was found of 1 min operating time and low microwave power level. While, the lowest value of extraction time (1.77 min) was found of 3 min operating time and high microwave power level.

Figure 10b shows the effect of ultrasonic temperatures (40, 60 and 80 °C) and operating time (15, 30 and 45 min) on extraction time from castor seeds. The results indicate that the extraction time increases with increasing ultrasonic temperature and operating time. It could be seen that the extraction time increased from 1.79 to 1.93, 1.92 to 2.11 and 2.00 to 2.21 min when the operating time increased from 15 to 45 min, respectively for 40, 60 and 80 °C ultrasonic temperature. The results also indicate that the highest value of extraction time (2.21 min) was found of 45 min operating time and 80 °C ultrasonic temperature. While, the lowest value of extraction time (1.79 min) was found of 15 min operating time and 40 °C ultrasonic temperature.

### Gas chromatography analysis

Table 1 shows the effect of pretreatment (microwave and ultrasonic) of castor seeds on the gas chromatography analysis compared to without pretreatment on the same conditions (200 °C extraction temperature and 60 rpm screw speed). The results indicate that, using of microwave for pretreatment of castor seeds was enhanced contents of oil yield properties more than those of ultrasonic pretreatment for castor seeds and without pretreatment. On the other hand, using of ultrasonic for pretreatment of castor seeds was enhanced quality of oil properties more than those of microwave pretreatment for castor seeds and without pretreatment. It could be seen that the Palmitic acid, Stearic acid, Oleic acid, Linolenic acid and α Linolenic acid were 1.26, 1.76 and 0.77, 1.35, 2.61 and 1.09, 4.47, 5.12 and 3.43, 5.41, 6.53 and 4.17 and 0.62, 0.54 and 0.49% for non-treatment, microwave pretreatment and ultrasonic pretreatment, respectively. These results agreed with those obtained by^23,24^ whose found that the castor oil is presents in minor amount that includes Stearic acid (1%), Linoleic acid (4.2%), Linolenic acid (0.3%), Dihydroxystearic acid (0.7%),Oleic acid (3.0%), Palmitic acid (1%), and Eicosanoic acid (0.3%)^17^.

**Table 1:** Effect of pretreatment of castor seeds on the gas chromatography analysis compared with non-treatment.

Item	Non-treatment	Pretreatment	
		Microwave	Ultrasonic
Palmitic acid (C16:0)	1.16	1.76	0.77
Stearic acid (C18:0)	1.35	2.61	1.09
Oleic acid (C18:1n9c)	4.47	5.12	3.43
Linoleic acid (C18:2n6c)	5.41	6.53	4.17
α, Linolenic acid (C18:3n3)	0.62	0.54	0.49
Ricinoleic acid (C18:1 OH)	86.99	83.44	90.05
Saponification No	179.9	178.7	182.4
Acid Value, %	1.56	2.11	1.13
Molecular weight (MW) of oil	943.70	953.06	928.45

The results also indicate that the Ricinoleic acid was 86.99, 83.44 and 90.05% for non-treatment, microwave pretreatment and ultrasonic pretreatment, respectively. These results agreed with those obtained by^6^ whose mentioned that the Ricinoleic acid is a major component of the castor seed oil where About 90% of the fatty acid content in castor oil is the triglyceride formed from Ricinoleic acid. It is an unsaturated omega-9 fatty acid and a hydroxy acid.

The results indicate that the acid values were 1.56, 2.11 and 1.13% for non-treatment, microwave pretreatment and ultrasonic pretreatment, respectively. Acid value is one of the important indicators of oil quality^26^. Omari et al*.*^27^ suggested that the high acid value of castor oil may be due to the delay in seed extraction which influenced the lipase enzyme to hydrolyze the triglycerides into free fatty acid^25^.

Saponification number values were 179.9, 178.7 and 182.4 for non-treatment, microwave pretreatment and ultrasonic pretreatment, respectively. These results agreed with those obtained by^7,25^. Also, the physicochemical properties of oil such as low acid value and free fatty acid percentage, high saponification value acid indicate that castor oil has good oil quality.

The molecular weight (MW) of oil value were 943.70, 953.06 and 928.45 for non-treatment, microwave pretreatment and ultrasonic pretreatment, respectively. The raw materials are converted into biodiesel through a chemical reaction involving alcohol and a catalyst. The specification of the molecular weight of crop oil is important for the biodiesel production process because the determination of the quantity of reactants is calculated according to the molecular weight of castor oil^28^. The results indicate that the lowest and the best of the molecular weight (MW) of oil value ultrasonic pretreatment then without pretreatment then microwave pretreatment.

## Conclusions

The experiment was carried out to study the effect of extraction screw speed (20, 40 and 60 rpm) and temperature (100, 150, 200 and 250 °C) on oil extraction yield from castor seeds, extraction energy and extraction time. Also, study the effect of pretreatment condition: microwave (low, medium and high power level and operating times were 1, 2 and 3 min) and ultrasonic temperature (40, 60 and 80 °C) and operating time (15, 30 and 45 min) for castor seeds before extraction at optimum conditions of screw press (200 °C extraction temperature and 60 rpm screw speed) on oil extraction yield from castor seeds, extraction energy, extraction time and component of oil production from extraction. The obtained results can be summarized as follows:

- The optimum extraction oil yield, extraction energy and extraction time were 35.59%, 18.68W.h and 1.86 min, respectively were found at 200 °C extraction temperature and 60 rpm screw speed.
- The oil extraction yield values were 32.67, 37.59 and 37.41% at low, medium and high microwave power levels, respectively, but they were 35.59, 36.37 and 37.08% for 40, 60 and 80 °C ultrasonic temperature, respectively.
- The extraction energy consumption were 20.0, 19.0 and 17.33 W.h for 1, 2 and 3 min microwave operating time, respectively, but they were 19.0, 20.3 and 21.0 W.h for 15, 30 and 45 min ultrasonic operating time, respectively.
- The highest value of extraction time (2.34 min) was found of 45 min operating time and 80 °C ultrasonic temperature. While, the lowest value of extraction time (1.77 min) was found of 3 min operating time and high microwave power level.
- The pretreatment improved properties of the extracted oil. But using microwave pretreatments had enhancement affect in oil properties better than that of the ultrasonic pretreatments and non-treatments. But, using of microwave pretreatment had enhancement better than that of ultrasonic pretreatment.

## Data Availability

The datasets used and/or analyzed during the current study available from the corresponding author on reasonable request.
